# Correction: Anthrax Toxin Receptor 2–Dependent Lethal Toxin Killing In Vivo

**DOI:** 10.1371/annotation/08c1bcf0-da65-4abd-97c7-5df86b1120b5

**Published:** 2008-10-16

**Authors:** Heather M Scobie, Darran J Wigelsworth, John M Marlett, Diane Thomas, G. Jonah A Rainey, D. Borden Lacy, Marianne Manchester, R. John Collier, John A. T Young

The authors also acknowledge support for this work from National Institutes of Health grant AI48489.

